# Correction: Signalling involving MET and FAK supports cell division independent of the activity of the cell cycle-regulating CDK4/6 kinases

**DOI:** 10.1038/s41388-020-1221-8

**Published:** 2020-02-24

**Authors:** Chi Zhang, Simon R. Stockwell, May Elbanna, Robin Ketteler, Jamie Freeman, Bissan Al-Lazikani, Suzanne Eccles, Alexis De Haven Brandon, Florence Raynaud, Angela Hayes, Paul A. Clarke, Paul Workman, Sibylle Mittnacht

**Affiliations:** 1 UCL Cancer Institute, University College London, London, WC1E 6DD UK; 2 Cancer Research UK Cancer Therapeutics Unit at The Institute of Cancer Research, London, SM2 5NG UK; 3 MRC Laboratory for Molecular Cell Biology, University College London, London, WC1E 6BT UK

**Keywords:** Checkpoint signalling, Growth factor signalling, Checkpoint signalling, Growth factor signalling, Growth factor signalling

**Correction to:Oncogene**

10.1038/s41388-019-0850-2

After publication of the article, the authors became aware of an error in Fig. 5e. In the published article, the immunoblot panels for p21CIP and SKIP2 for cell lines MCF7 and A549 in Fig. 5e were interchanged. A correct version of the Figure is published below. This has now been corrected in both the PDF and HTML versions of the article.

**Figure:**
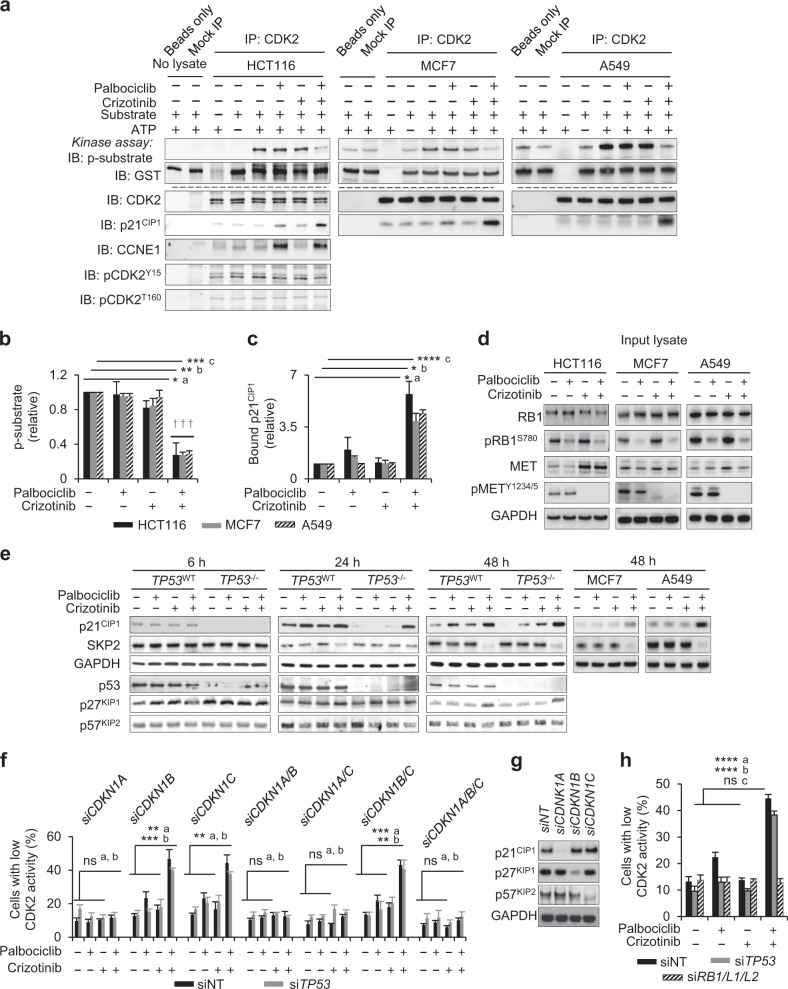
Fig. 5

