# Supplementary figures and images for: Signalling involving MET and FAK supports cell division independent of the activity of the cell cycle-regulating CDK4/6 kinases
Source: Oncogene. 2019 Jul 12;38(30):5905–20. doi: 10.1038/s41388-019-0850-2 (PMC6756076; doi:10.1038/s41388-019-0850-2)

Figure S1

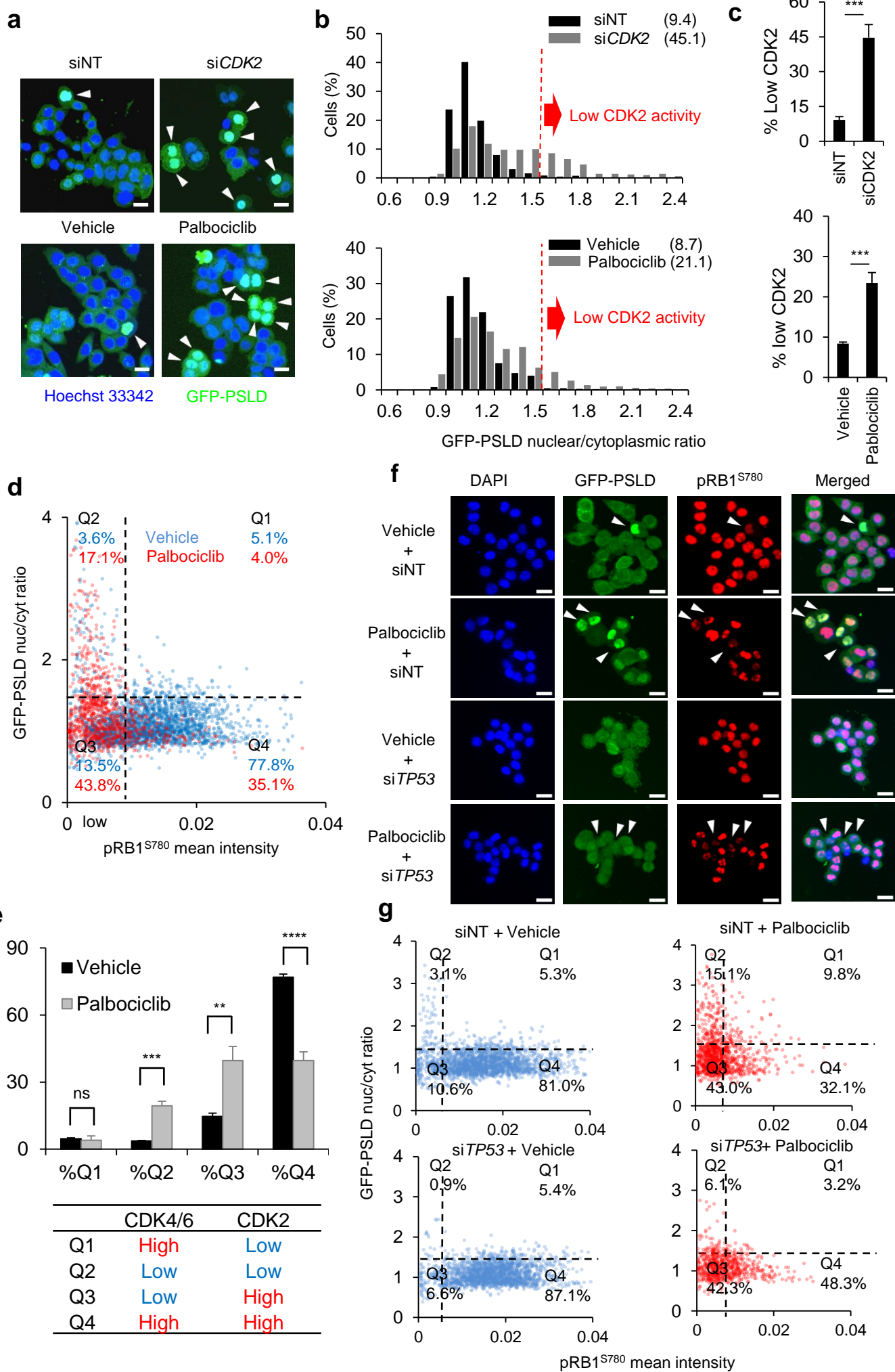

Figure S2

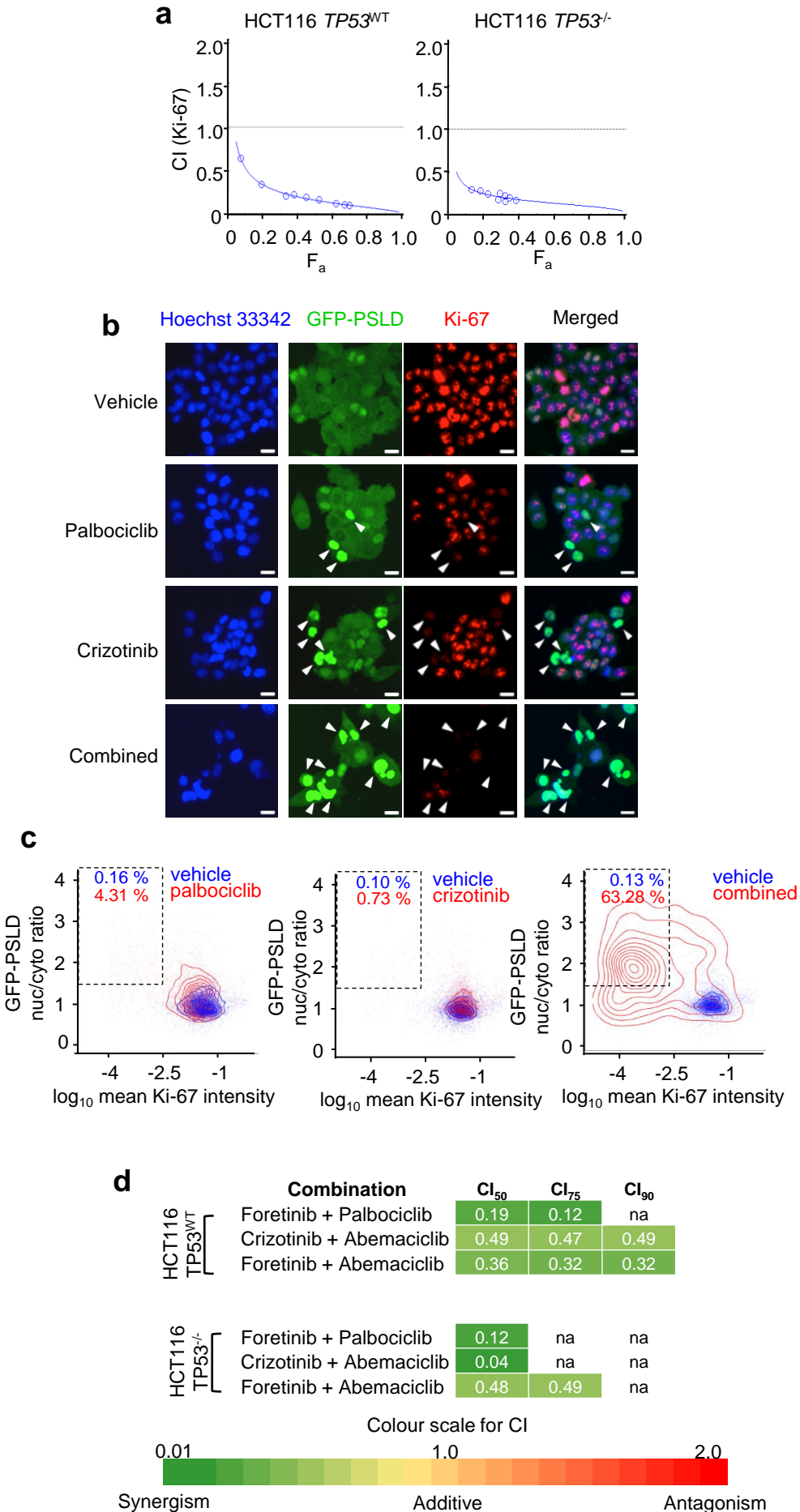

Figure S3

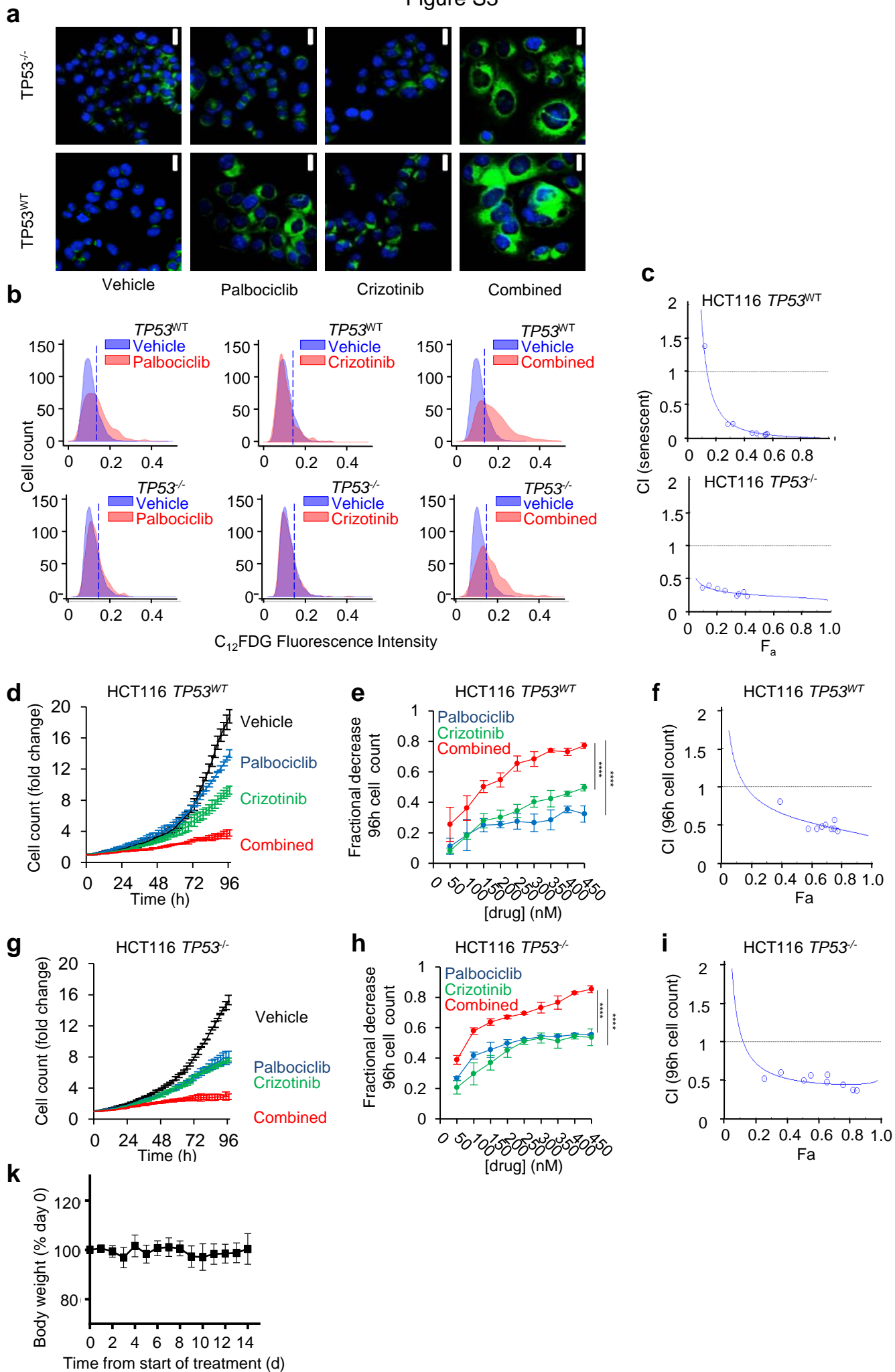

Figure S4

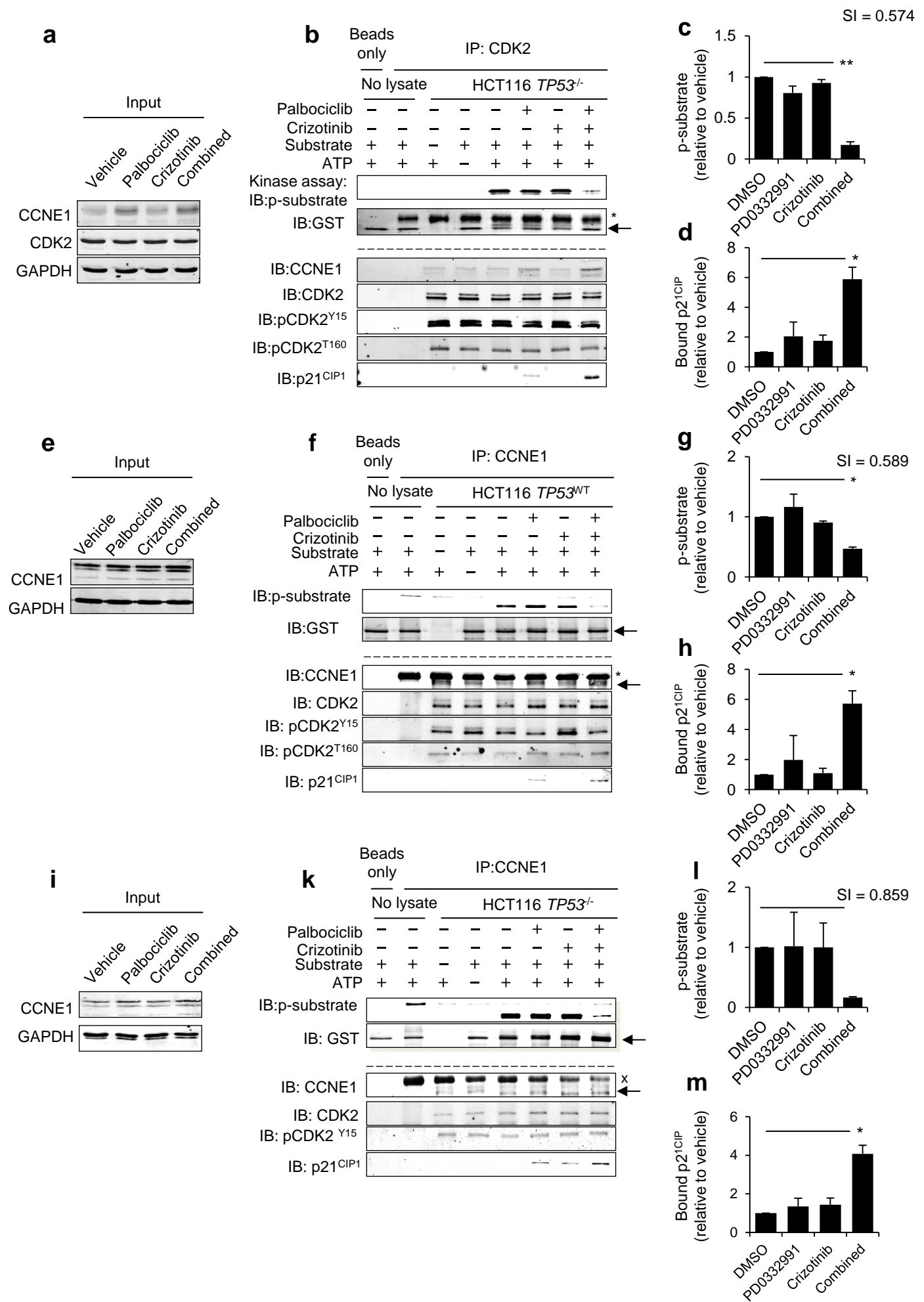

Figure S5

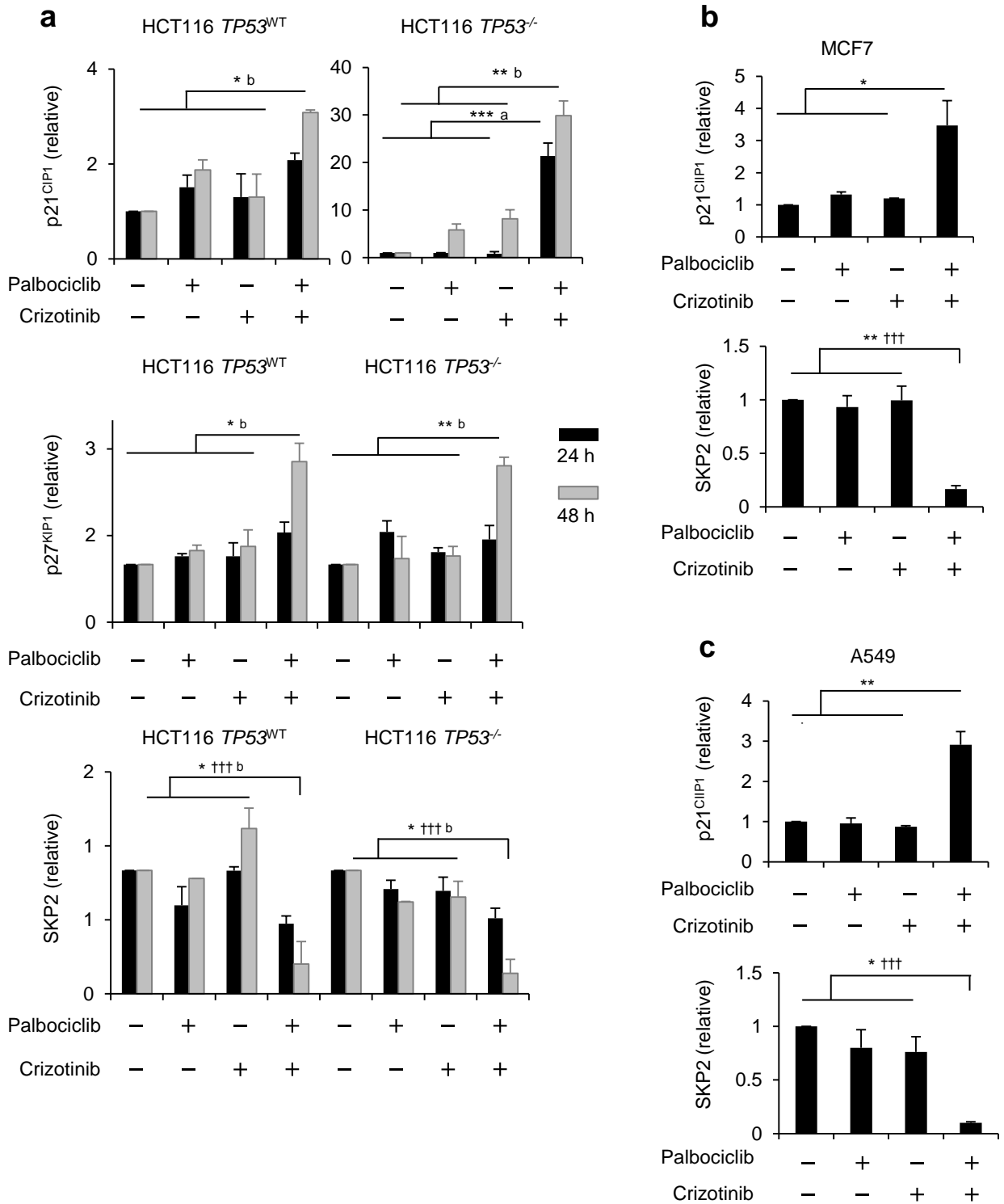

Figure S6

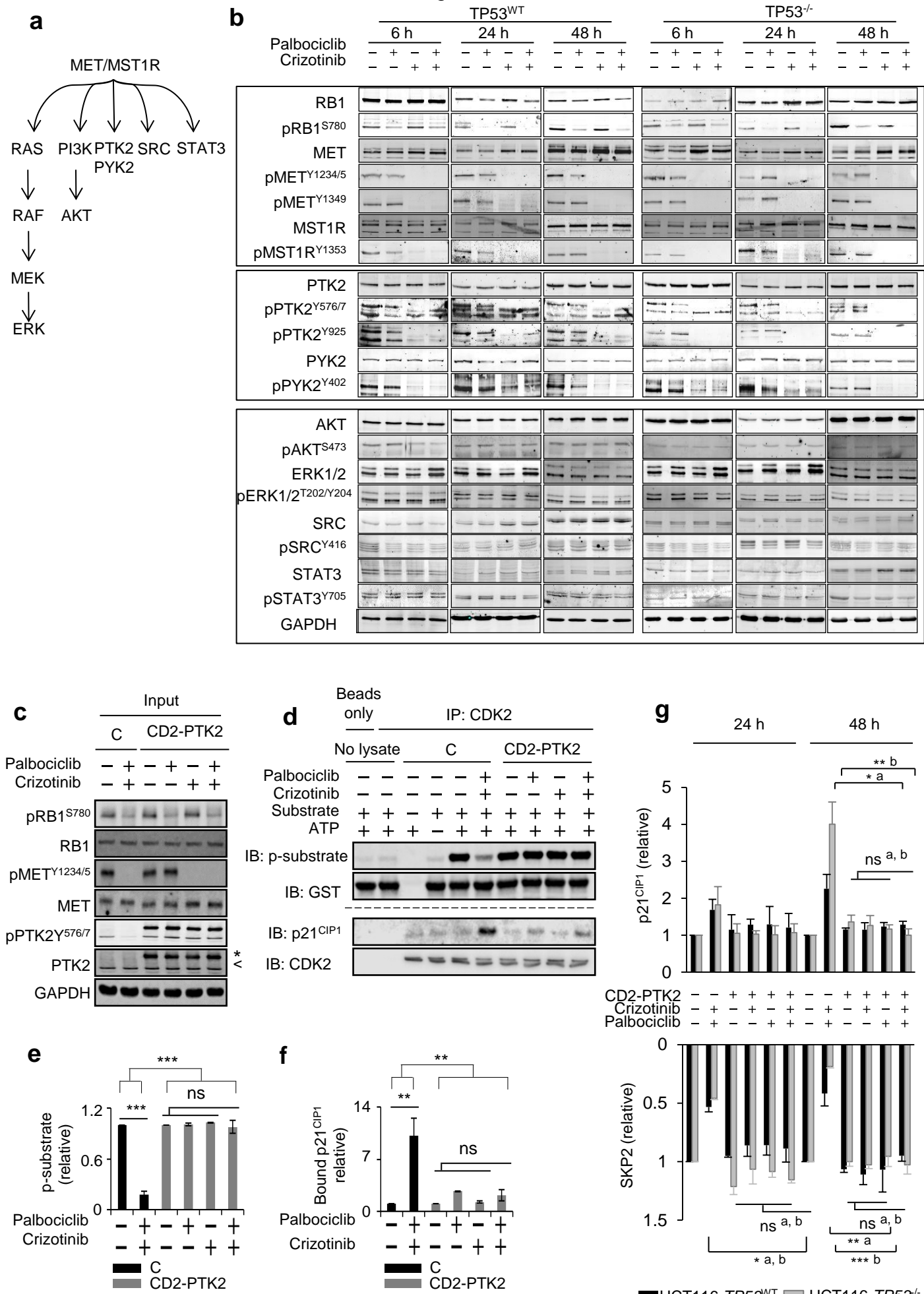

Figure S7

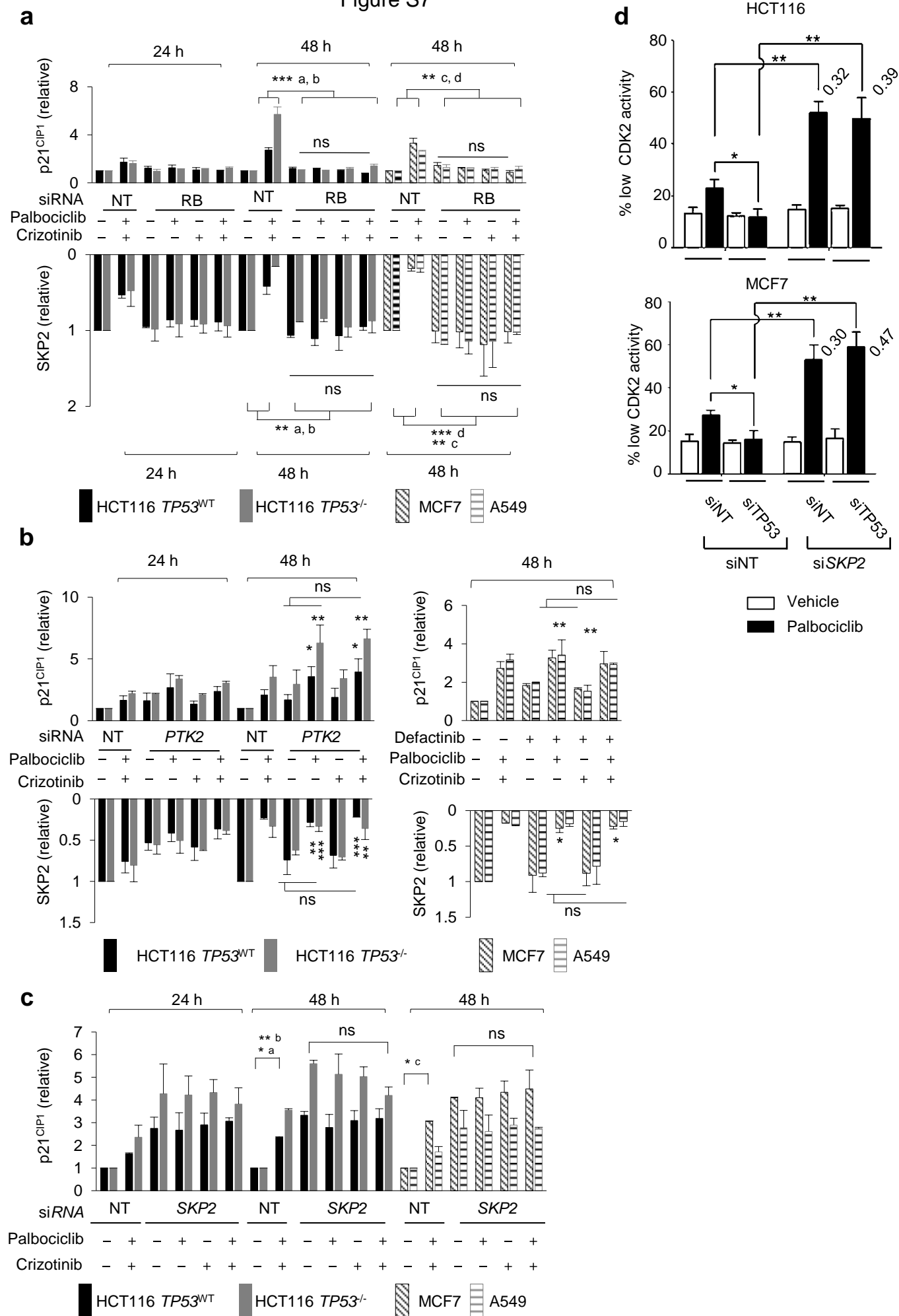

Supplement: Supplementary file 2 — Supplementary figures S1-S7 [file 41388_2019_850_MOESM2_ESM.pdf]
