# Supplementary material for: Signalling involving MET and FAK supports cell division independent of the activity of the cell cycle-regulating CDK4/6 kinases
Source: Oncogene. 2019 Jul 12;38(30):5905–20. doi: 10.1038/s41388-019-0850-2 (PMC6756076; doi:10.1038/s41388-019-0850-2)
Supplement: Supplementary file 3 — Supplementary tables S1-S2 [file 41388_2019_850_MOESM3_ESM.pdf]

Supplemental Table S1: Results of MetaCore™ pathway map enrichment analysis using hits identified in *TP53*$^{-/-}$ cells.

Rank	Name of MetaCore™ pathway map	No. of kinases in pathway	No. of hits	p-value	Hits mapped
1	MET and MSP receptor (RON) signalling pathways in SCLC	18	5	3.00×10^{-5}	*MST1R, PTK2B, MET, CDK2, PTK2*
2	SDF-1 signalling in hematopoietic stem cell homing	11	3	1.73×10^{-3}	*PTK2B, PTK2, JAK2*
3	The role of PTEN and PI3K signalling in melanoma	13	3	2.92×10^{-3}	*BRAF, PTK2, JAK2*
4	HBV-dependent NF-kB and PI3K/AKT pathways leading to HCC	13	3	2.92×10^{-3}	*PTK2B, CDK2, PTK2*
5	FGFR3 signalling in multiple myeloma	14	3	3.66×10^{-3}	*PTK2B, FGFR3, JAK2*
6	Resolution of inflammation in healing myocardial infarction	5	2	5.34×10^{-3}	*MET, JAK2*
7	Proliferative action of gastrin in pancreatic cancer	16	3	5.44×10^{-3}	*PTK2B, PTK2, JAK2*
8	CXCR4 signalling pathway	16	3	5.44×10^{-3}	*PTK2B, PTK2, JAK2*
9	Growth hormone signalling via PI3K/AKT and MAPK cascades	17	3	6.54×10^{-3}	*PTK2B, PTK2, JAK2*
10	Cadherin-mediated cell adhesion	6	2	7.91×10^{-3}	*MET, PTK2*

Supplemental Table S2: Results of MetaCore™ pathway map enrichment analysis using hits identified in *TP53*^{WT} cells.

Rank	Name of MetaCore™ pathway map	Total kinases in pathway	No. of hits	p-value	Hits mapped
1	Role of SCF complex in cell cycle regulation	9	4	7.53×10^{-5}	*PLK1, CDK2, CKS1B, CDK1*
2	Role of APC in cell cycle regulation	9	4	7.53×10^{-5}	*PLK1, CDK2, CKS1B, CDK1*
3	Cell cycle progression in prostate cancer	11	4	1.89×10^{-4}	*CDK2, RPS6KB1, CDK1, JAK2*
4	nNOS signalling in neuronal synapses	2	2	9.08×10^{-4}	*DLG4, CALM3*
5	Abnormalities in cell cycle in SCLC	9	3	1.88×10^{-3}	*CDK2, CKS1B, CDK1*
6	Leptin signalling in colorectal cancer	10	3	2.63×10^{-3}	*RPS6KB1, CDK1, JAK2*
7	Constitutive and activity-dependent synaptic AMPA receptor delivery	10	3	2.63×10^{-3}	*DLG4, DLG1, CALM3*
8	Transition and termination of DNA replication	3	2	2.67×10^{-3}	*CDK2, CDK1*
9	Main chemotherapy drugs and their action in SCLC cells	24	4	4.71×10^{-3}	*ABL1, CDK2, RPS6KB1, CDK1*
10	Role of nicotine-induced leptin resistance in hypothalamus in development of obesity	4	2	5.25×10^{-3}	*RPS6KB1, JAK2*
